# Convenient colorimetric approach to quantify CuO NPs in water using only a smartphone and cellulose paper with an immobilised chemosensor

**DOI:** 10.1007/s00604-025-07439-9

**Published:** 2025-08-16

**Authors:** Jesús Sanmartín-Matalobos, Pilar Bermejo-Barrera, Ana M. García-Deibe, Matilde Fondo, Yeneva Alves-Iglesias

**Affiliations:** 1 Institute of Materials (iMATUS), Universidade de Santiago de Compostela, Avenida do Mestre Mateo 25, 15782 Santiago de Compostela, Spain; 2 Coordination and Supramolecular Chemistry Group (SupraMetal), Department of Inorganic Chemistry, Faculty of Chemistry, Universidade de Santiago de Compostela, Avda. das Ciencias S/N, Campus Vida, 15782 Santiago de Compostela, Spain; 3 Trace Element, Speciation and Spectroscopy Group (GETEE), Department of Analytical Chemistry, Nutrition and Bromatology, Faculty of Chemistry, Universidade de Santiago de Compostela, Avenida das Ciencias S/N, Campus Vida, 15782 Santiago de Compostela, Spain

**Keywords:** CuO NPs, Chemosensor, Fluorescence, Diffuse reflectance, Smartphone assisted detection

## Abstract

A novel approach is reported to quantify CuO NPs in aqueous samples by using digital image colorimetry (DIC), which is a rapid, low-cost, and facile method. We have used a colour recogniser application on a smartphone to register the gradual colour changes experimented, under *λ*_ex_ = 365 nm radiation, by a dansyl-based chemosensor immobilised on cellulose paper, after interacting with aqueous dispersions of CuO NPs with concentrations between 5 and 100 µg L^−1^. The low limits of detection (LOD) and quantification (LOQ) (10.5 and 34.9 µg L^−1^, respectively) show a relevant sensitivity for this simple method, which also displays a good precision and accuracy (analytical recovery, 99 ± 1%). A comparison of the results similarly obtained for the modified paper through diffuse reflectance (DR) revealed worse detection parameters (LOD = 100.0 µg L^−1^ and LOQ = 333.4 µg L^−1^). In addition, fluorescence spectroscopy (*λ*_em_ = 520 nm) demonstrated the ability of the free chemosensor (1.26 µg L^−1^ in 20:80 (v/v) water–ethanol solutions) for the quantification of CuO NPs (LOD = 30 µg L^−1^ and LOQ = 101 µg L^−1^), without improvement of the results obtained with DIC on the modified papers. The dissolved free chemosensor demonstrated selectivity towards CuO NPs in the presence both of metal ions common in aqueous samples as K^+^, Na^+^, Ca^2+^, Mg^2+^, Fe^3+^, or Al^3+^ and of other usual nanomaterials as those of Cu, CdSe, TiO_2_, and ZnO NPs.

**Graphical Abstract:**

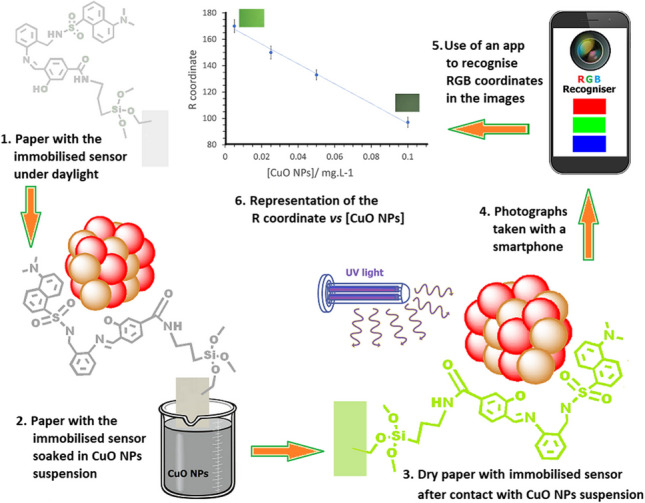

**Supplementary Information:**

The online version contains supplementary material available at 10.1007/s00604-025-07439-9.

## Introduction

The remarkable chemical and physical properties of CuO nanoparticles (NPs), including chemical stability, excellent electrochemical activity, and superthermal conductivity, have led to their extensive use in diverse fields of health [1–3], industry [4–8], and environment [9–13]. However, this widespread use of CuO NPs is not without health hazards [14–19] and environmental risks [20–22], as studies have reported that CuO NPs are considered toxic at any concentration above 1 mg/L. Both in vitro as well as in vivo studies point out that CuO NPs can induce oxidative stress, inflammation, neurotoxicity, immunotoxicity, cytotoxicity, and genotoxicity in bacteria, algae, rodents, fishes, and human cell lines [23, 24]. The main toxicological mechanisms of CuO NPs can originate reactive oxygen species (ROS), inducting oxidative stress [25]. Thus, Assadian and col. [26] have shown that exposure to these nanoparticles can be related to suppression of the immune system in humans, because of the induction of oxidative stress in lymphocytes that can lead to cell death.

Accurate detection and monitoring of CuO NPs are therefore essential for assessing environmental exposure, ensuring regulatory compliance, and guiding the safe and sustainable development of nanotechnology. As a result of these worries, the enhancement of analytical methods focused on the detection of CuO NPs is rising a growing interest. In this way, the use of techniques as single particle ICP-MS [27–32], ICP-OES [33], and microscopy [34–36] has been reported for characterisation and quantification of CuO NPs in plants, soil, natural waters, and cells. Among others, Navratilova et al. [27] utilised single particle ICP-MS for detecting CuO NPs in colloidal samples extracted from soils. Peng et al. have employed ICP-OES to quantify the presence of copper on rice roots grown in soils with added CuO NPs [33]. Likewise, Zhao et al. [34] have utilised EDS analyses to measure the occurrence of copper on roots of aquatic florae exposed to CuO NPs. Likewise, in recent years, we have reported the use of rapid and sensitive optical methods to measure the concentration of CuO NPs in solution, through decreases in the fluorescence emission of tosyl-based fluorescent probes [37, 38]. Now, we are focused on DIC using smartphones as a rapid, low-cost, and facile, but powerful analytical tool to measure colour changes related to a target analyte, using digital images captured by their built-in cameras [39–41]. The RGB model is one of the most used systems to describe colours in a 3D coordinate space, as each colour is expressed as a single point.

Here, we report an innovative colorimetric approach to quantify CuO NPs in water using only a smartphone, and cellulose paper with an immobilised chemosensor. We present the development of a dual-mode chemosensor that integrates both colorimetric and fluorescence detection, offering enhanced sensitivity and analytical reliability. This sensing platform is further strengthened by the incorporation of smartphone-based quantification and image analysis, which allows for real-time, on-site detection without the need for sophisticated instrumentation. Additionally, we demonstrate a simple, low-cost fabrication process that is not only accessible and reproducible but also holds strong potential for scalable production and field deployment. These features collectively differentiate our approach from previous work and underline the practical relevance and innovation of the proposed sensing platform.

Cellulose-based chemosensors have garnered considerable attention in recent years for environmental monitoring [42–49]. Their key advantages include low cost and availability, portability and flexibility, no need for external power, integration with mobile devices, minimal reagent consumption, biodegradability and eco-friendliness, ease of functionalisation, and rapid and visual detection. However, limited sensitivity and selectivity, short shelf life and stability issues, environmental interferences, standardisation and calibration, single-analyte focus, data interpretation and integration, and mechanical fragility still hinder the broader application of paper-based chemosensors for environmental monitoring.

An unsophisticated smartphone was used not only to take photographs under UV light of the fluorescent emission changes displayed by this modified paper, but also to detect their average RGB values with a free colour recognition app. For this purpose, we have selected a previously synthesised dansyl-based fluorescent probe [50], which can be covalently immobilised on cellulose paper [51]. The free ligand displays a suitable *N,N,O* donor set, in joint with a sulphonamide group, which can effectively bind unsaturated Cu^2+^ ions existing on the surface of the NPs.

Diffuse reflectance (DR) and fluorescence spectroscopy measurements were also carried out to compare the accuracy of the smartphone-based DIC for detecting CuO NPs, with these two classic determination methods. The DR studies correlated the decrease of a characteristic absorption band (*λ*_abs_ = 270 mm) of the chemosensor immobilised on cellulose paper, with increasing concentrations of CuO NPs. In addition, we studied the decline in the fluorescence intensity (*λ*_em_ = 530 nm) of the free chemosensor dissolved in ethanol–water solutions with growing concentrations of CuO NPs. Studies on potential interferences with this determination procedure were also performed in ethanol–water solutions, not only using several other ordinary nanomaterials, but also some metal ions habitually present in water samples.

## Materials and methods

### List of reagents

The list of reagents is as follows: Whatman® qualitative filter paper discs 55-mm diameter, Grade 2 (Sigma-Aldrich), (3-aminopropyl)trimethoxysilane (APTMS), 97% (Sigma-Aldrich); *N*-hydroxysuccinimide (NHS), 98% (Sigma-Aldrich); *N*-(3-dimethylaminopropyl)-*N*-ethylcarbodiimide hydrochloride (EDC·HCl), 98% (Sigma-Aldrich); *N*,*N*-dimethylformamide (DMF), ≥ 99.8%, anhydrous (Sigma-Aldrich); triethylamine (TEA), ≥ 99% (Sigma-Aldrich), Ultrapure Milli-Q® water, ethanol absolute (Sigma-Aldrich), ZnO NPs ≤ 40 nm (Sigma-Aldrich), TiO_2_ NPs < 150 nm (Sigma-Aldrich); Cu NPs 40–60 nm (Sigma-Aldrich); CuO NPs < 50 nm (Sigma-Aldrich), KCl, 99.0% (Sigma-Aldrich); NaCl, 99.0% (Sigma-Aldrich); CaCl_2_, ≥ 99% (Sigma-Aldrich); MgCl_2_, ≥ 98% (Sigma-Aldrich); FeCl_3_, 97% (Sigma-Aldrich); Al(NO_3_)_3_·9H_2_O, ≥ 98% (Sigma-Aldrich).

### Immobilisation of the chemosensor on cellulose paper

**Fig. 1:**
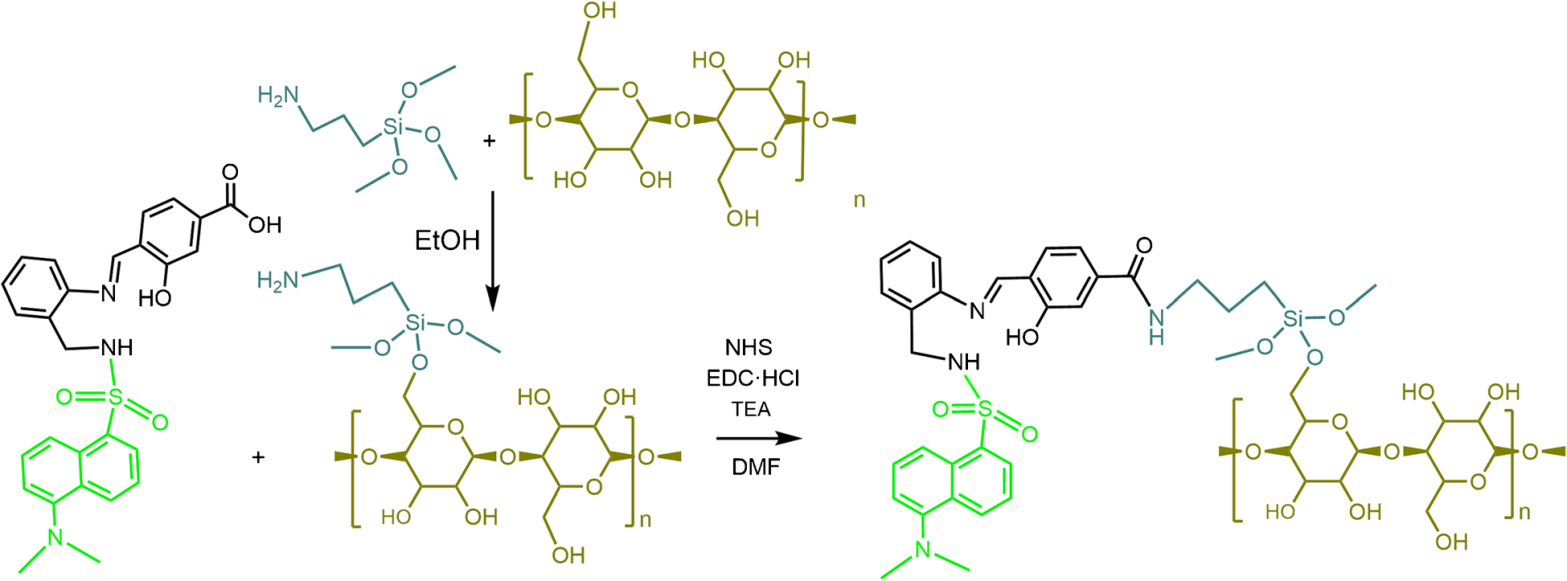
Schematic representations of the procedure for covalent immobilisation of the chemosensor on cellulose paper. The dansyl antenna is highlighted in light green, and the part related to the immobilisation on the cellulose polymer (olive green) has been bluish grey coloured

The description of the synthesis and subsequent characterisation of the chemosensor used herein has been previously reported [50]. Figure 1 shows a schematic illustration of the procedure for its covalent immobilisation on cellulose paper [51].

A cellulose filter paper (55-mm diameter) was immersed in a 1 wt.% ethanolic solution of APTMS for 1 h at room temperature. The resulting amine-functionalised cellulose paper was thoroughly washed with ethanol and dried under vacuum at 40 °C. To activate the carboxyl group of the chemosensor for amide bond formation with amine-functionalised cellulose paper, equimolar amounts of NHS and EDC·HCl (0.1 mmol each) were added to a DMF solution (20 mL) containing the chemosensor (0.1 mmol). The mixture was stirred at room temperature for 3 h, followed by the addition of TEA (0.3 mmol). The activated chemosensor solution remains stable and effective for up to 48 h after preparation; beyond this point, its suitability for functionalisation is compromised. The amine-modified cellulose paper was then immersed in the chemosensor solution and left to react overnight. Finally, the chemosensor immobilised on cellulose paper was washed sequentially with water and ethanol and dried under vacuum at 40 °C.

Diffuse reflectance (DR) and scanning electron microscopy-energy dispersive X-ray spectroscopy (SEM–EDX) were used to characterise the chemosensor papers. The DR spectra of the chemosensor papers were registered on a LAMBDA 1050 + UV/Vis/NIR PerkinElmer spectrophotometer, which was equipped with an integrating sphere. SEM images and EDX spectrum were obtained using a ZEISS FESEM ULTRA Plus with EDX.

### Smartphone-based DIC

#### Sample preparation

Discs of the chemosensor-modified cellulose paper were immersed for 15 min in water dispersions of CuO NPs with concentrations of 5, 25, 50, and 100 µg L^−1^. No pH-adjusting agents were added. To ensure homogeneity, all samples were sonicated prior to measurement, thereby improving accuracy and reproducibility. Each suspension was sonicated for 5 min prior to paper immersion. Prior to being measured, all the soaked discs of cellulose paper had been conveniently air-dried. For each assay, at least three replicates were made for statistical purposes.

#### Image acquisition and color data standardisation

The air-dried paper discs were placed in an UV viewing booth (C-10E4 Mini UV Cabinet, Analytik Jena), under light of 365 nm wavelength, and then photographed with a smartphone to digitalise their chromatic information. The use of a UV viewing booth, with a smartphone mounted in the viewport, largely prevents daylight interference when taking photos.

Photographs of the chemosensor-modified paper were captured with the integrated camera (50 MP) of a Samsung Galaxy A25 smartphone running Android 14 OS. The same camera settings were used for all photographs (white balance, light sensitivity, etc.). HDR (High Dynamic Range) was enabled, and no filters or image optimisers have been used. Higher smartphone camera resolution enhances the detail and accuracy of the RGB colour measurements.

The free application RGB Color Detector [52], for Android OS 9.0 or up, was used to calculate the RGB coordinates. RGB Color Detector is a free application available for Android and macOS that provides access to a wide range of colour formats and conversions, including RGB, CMYK, HSV, HTML, HEX, and HSL. The software analyses images and extracts the RGB values of each pixel. This colour analysis enables the calculation of linear regressions based on colour data.

Colour information was obtained from three replicate photographs, each taken at five distinct points across different areas of the paper samples following immersion in aqueous dispersions of CuO NPs. To enable response correction, measurements were also performed on the chemosensor-modified cellulose paper prior to exposure to the CuO NP dispersions. The raw RGB values extracted from the images were normalised and processed prior to any calibration. This process included background subtraction and correction for ambient light conditions. A reference colour chart (including white and black points) was included in all images to allow for image-to-image normalisation. This mitigated the effect of baseline variation due to lighting, device camera, or environmental changes. Calibration curves were generated with standardised imaging conditions, and measurements were repeated across multiple days. The results showed minimal baseline shifts, which have been considered. When necessary, calibration curves were adjusted to reflect small inter-day variations.

### UV–Vis-NIR measurements

#### Sample preparation

Four tap water suspensions of CuO nanoparticles (20 mL each) were prepared at concentrations of 100, 350, 500, and 1000 µg·L⁻^1^. No pH-adjusting agents were added. Each suspension was sonicated for 5 min prior to paper immersion. Chemosensor-modified cellulose paper discs were then immersed in the freshly prepared CuO NP suspensions for 2 h. After immersion, the discs were air-dried before measurement.

The diffuse reflectance (DR) spectra of the paper discs were registered on a LAMBDA 1050 + UV/Vis/NIR PerkinElmer spectrophotometer, which was equipped with an integrating sphere.

### Fluorescence quenching measurements

#### Sample preparation

Ethanol–water solutions (80:20, v/v) of 4 mL were prepared containing the free chemosensor (1.26 µg·L⁻^1^), NaOH (0.18 µg·L⁻^1^), and CuO NPs at concentrations of 16, 32, 64, 125, 187, and 250 µg·L⁻^1^. Prior to being measured, each suspension was sonicated for 5 min.

Fluorescence emission studies of ethanol–water solutions were accomplished on a Shimadzu RF-600 Spectro Fluorophotometer.

## Results and discussion

The synthesis and characterisation of the chemosensor used in this study have been previously reported [50]. Based on this groundwork, we performed its covalent immobilisation onto cellulose paper [51] using a procedure detailed in the experimental section. This enables stable integration of the sensing element into the paper matrix and ensures its functionality within the analytical platform.

Before initiating the study on the determination of CuO NPs in laboratory-prepared samples using smartphone-based DIC, the immobilisation procedure of the chemosensor onto cellulose paper was first optimised and evaluated.

### Immobilisation of the chemosensor on cellulose paper

Since sensor immobilisation involves immersion in an activated DMF solution composed of the chemosensor, NHS, EDC·HCl, and TEA, we examined the temporal stability of this solution and assessed how long it remains effective for reuse. The stability and reactivity of the activated chemosensor solution were evaluated by monitoring changes in the diffuse reflectance spectra over time. Four cellulose papers were functionalised using the same solution from its initial preparation up to 72 h later (at 0, 24, 48, and 72 h). As shown in Figure S1 (SI), the diffuse reflectance spectra of the three chemosensor-treated papers prepared during de first 48 h remained unchanged, with three characteristic absorption bands at approximately 270, 330, and 420 nm. This evidence shows that both the solution and the immobilised species maintain their chemical integrity throughout this period. In contrast, spectral alterations observed at 72 h suggest chemical degradation or transformation of the active species. These results indicate that the chemosensor solution remains stable and effective for up to 48 h after preparation; beyond this point, its suitability for functionalisation is compromised.

### Characterisation of the immobilised chemosensor

**Fig. 2:**
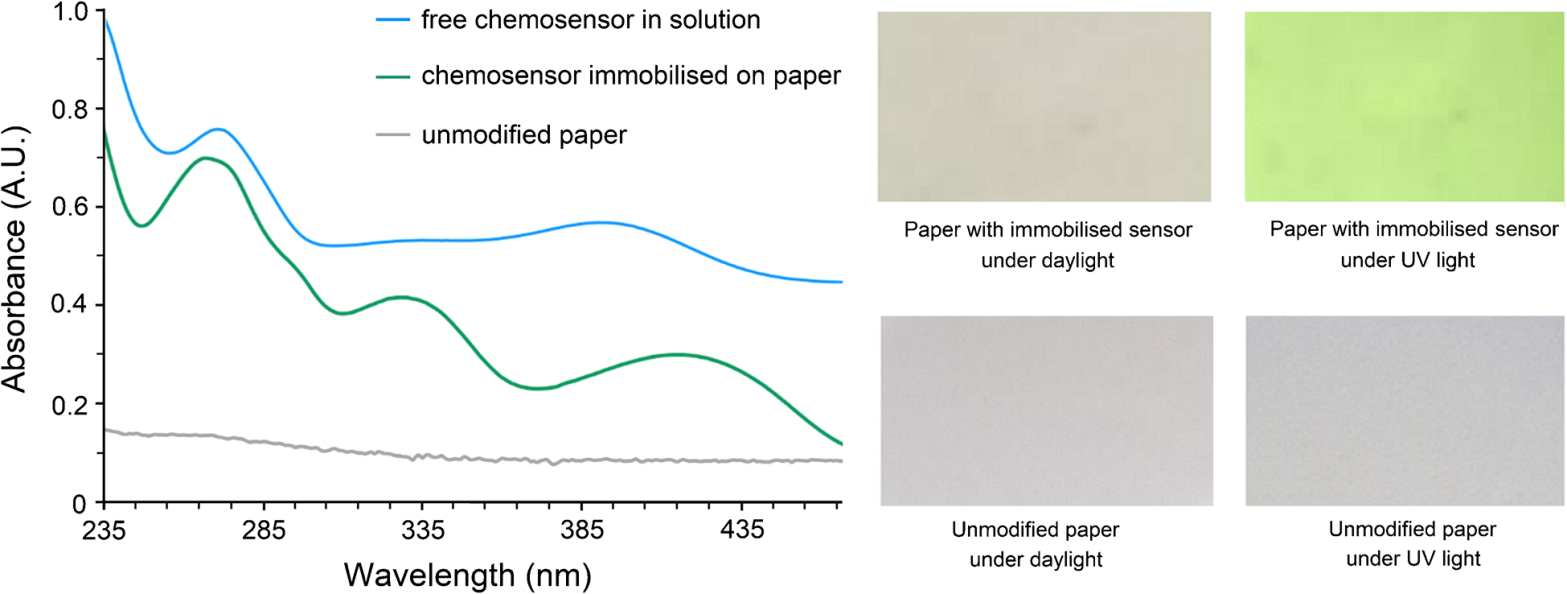
Left: View of the diffuse reflectance spectra corresponding to the unmodified cellulose paper as a grey line, while the green one corresponds to the chemosensor immobilised on it. The UV–Vis spectrum of the free chemosensor dissolved in an 80:20 ethanol:water mixture (v/v) is shown as a blue line. Right: Photographs of the unmodified paper and the chemosensor paper under daylight and UV light

We employed a combination of DR and UV–Vis spectroscopies, as well as fluorescent imaging and SEM–EDX, to confirm the immobilisation of the chemosensor on cellulose paper. Immobilisation of the chemosensor induces a subtle colour change in the cellulose paper (from white to pale grey), which is visible to the naked eye (Fig. 2, right). Furthermore, when this modified paper is placed under an ultraviolet light source of wavelength 254 nm, it displays a bright clear green colour. The uniform fluorescence emission observed provides preliminary evidence of the successful immobilisation of the chemosensor on the paper substrate (Fig. 2, right).

The DR spectrum of the chemosensor anchored on cellulose paper (green line, Fig. 2) exhibits characteristic absorption bands at approximately 270, 330, and 420 nm, attributed to π-π* electronic transitions [50]. This is in evident contrast with the DR spectrum of the cellulose paper (grey line, Fig. 2), which does not show bands in the range 235–450 nm. A comparison between the spectra of the chemosensor in solution (blue line, Fig. 2) and anchored on cellulose reveals that the 330 nm band is less distinguishable in solution, due to its broader profile and lower intensity. Increasing the concentration of the chemosensor solution makes the band at 330 nm more clearly observable. The absorption band centered at 390 nm in the dissolved free chemosensor exhibits a red shift of approximately 30 nm upon immobilisation of the chemosensor on cellulose paper, which is attributed to the formation of the amide bond. The absence of absorption bands in the UV–Vis spectrum of the washing water from the chemosensor-functionalised cellulose paper indicates that the chemosensor does not leach into the solution and remains bound to the cellulose matrix. These findings confirm the occurrence of an interaction between the chemosensor and the cellulose paper.

SEM–EDX analysis confirmed the successful integration of the chemosensor, as evidenced by the detection of S, Si, and N within the cellulose matrix fibres (Figure S2, SI). It must be noted that the Kα lines of carbon (0.277 keV) and oxygen (0.525 keV) are located in close proximity to the nitrogen Kα line (0.393 keV), which complicates the accurate identification and quantification of nitrogen. SEM analysis indicated no morphological changes in the cellulose fibres post-chemosensor interaction, with diameters between 10 and 20 μm.

### Fluorescence and colorimetric characterisation of the chemosensor

3D fluorescence spectra of the chemosensor in an ethanol:water mixture (80:20) were recorded to explore all excitation/emission wavelength combinations and determine the excitation wavelength that produces the maximum emission intensity. The use of an ethanol–water mixture not only enhances the sensor’s fluorescence emission intensity, owing to its polarity, but also serves as an appropriate medium for dissolving the sensor and effectively dispersing the CuO NPs under investigation. Excitation wavelength versus fluorescence wavelength 3D fluorescence spectra have been obtained by successively varying the excitation wavelength as fluorescence spectra were measured. Analysis of the 3D fluorescence spectra revealed that the chemosensor dissolved in an ethanol:water (80:20) mixture absorbs UV light at approximately 400 nm and exhibits a strong green fluorescence emission centered around 520 nm (Figure S3, SI).

The fluorescence response of the chemosensor was further studied as a function of pH. An increase in fluorescence intensity was observed with rising pH, which is consistent with the progressive deprotonation of the molecule. Monodeprotonation occurs at pH values below 7, attributed to the carboxylic acid group with a pKa of approximately 4. Further deprotonation of the phenol and sulfonamide groups requires higher pH values, as their pKa values are around 9. As a result, a 2.5-fold increase in fluorescence intensity was observed at pH 9 compared to that at near-neutral pH.

Studies on the fluorescence stability of an EtOH:H_2_O (80:20) solution of the chemosensor were carried out under controlled environmental conditions to evaluate its performance over time. Aliquots of the sensor solution were stored in the dark at 5 °C, and fluorescence intensity was measured after 1, 3, 5, and 7 days to assess its stability under storage conditions. The results showed that the fluorescence emission intensity remained stable with no significant changes over a 5-day period. However, after 7 days, an increase in fluorescence intensity was observed, which was attributed to the hydrolysis of the chemosensor. It is important to note that the hydrolysis of the imino group yields *N*-(2-aminobenzyl)−5-(dimethylamino)naphthalene-1-sulfonamide, a compound that exhibits higher fluorescence emission at 520 nm than the original chemosensor.

The colour stability of the chemosensor paper was evaluated by monitoring DR spectral changes over time. Given the potential susceptibility of the chemosensor paper to environmental factors such as humidity, temperature fluctuations, light, and dust, the paper-based chemosensors were stored in dry, dark conditions with controlled and stable temperature. The absence of significant spectral shifts or intensity changes compared to the freshly prepared chemosensor paper indicates that the sensor remains stable and effective for up to 6 months after immobilisation. Digital analysis of photographs taken from the chemosensor paper further confirms the absence of significant colour changes. Extended exposure to UV light leads to significant bleaching of the chemosensor paper within 6 h. After this period, noticeable colour changes occur, and by 12 h, the paper exhibits yellowing, indicating a loss of sensor functionality.

### DIC for CuO NP detection

Prior to CuO NP detection via DIC, a preliminary investigation was performed to identify the optimal pH and immersion time needed for stabilization of the R coordinate signal when cellulose paper is exposed to a CuO NP dispersion.

#### Time of immersion for DIC

Six paper samples were immersed in an aqueous solution of CuO NPs at a concentration of 20 mg·L⁻^1^. The immersion times evaluated were 15, 30, 45, 60, and 120 min, as well as overnight. After each time interval, the papers were removed from the solution and allowed to air dry. The variation in the R colour coordinate was analysed using digital imaging under 365 nm. Figure S4 (SI) shows that the R coordinate stabilises after 15 min of immersion.

#### Selection of pH for DIC

When selecting the pH values, we considered that the chemosensor contains –OH and -SO_2_(NH)- functional groups with pKa values around 9. At pH = 9, these groups are partially or fully deprotonated, which facilitates electrostatic interactions with the CuO surface. In the absence of pH modifiers, the chemosensor remains almost completely protonated, which affects the strength of the interaction, although the same functional groups may still be involved. Since pH values below 7 led to partial dissolution and aggregation of CuO NPs, and values above 9 may cause chemical instability through the formation of Cu(OH)_2_ or soluble complexes, measurements were conducted at pH values near neutrality and at pH 9.

To assess the effect of pH on the interaction between the chemosensor and CuO NPs, cellulose papers functionalised with the chemosensor were submerged overnight in two separate aqueous suspensions of CuO NPs (300 mg·L⁻^1^), one of the suspensions without added pH modifiers and the other adjusted to pH = 9. Then, the papers were retrieved and air-dried at room temperature.

A straightforward visual inspection indicated that the suspension at pH = 9 developed a yellowish coloration (indicative of chemical reactivity), accompanied by a loss of homogeneity in the CuO nanoparticle dispersion due to the formation of aggregates. Conversely, no noticeable colour change occurs in the suspension without added pH modifiers, which remains homogeneous throughout. Using DR spectroscopy, we observed that the intensity of the bands at 270 and 420 nm is twice as low in the spectrum of the paper immersed in the suspension without added pH modifiers, indicating a higher concentration of CuO NPs. Based on these findings, no pH-adjusting agents were added to the suspension for CuO NP detection.

#### Smartphone-based colorimetric detection of CuO NPs

**Fig. 3:**
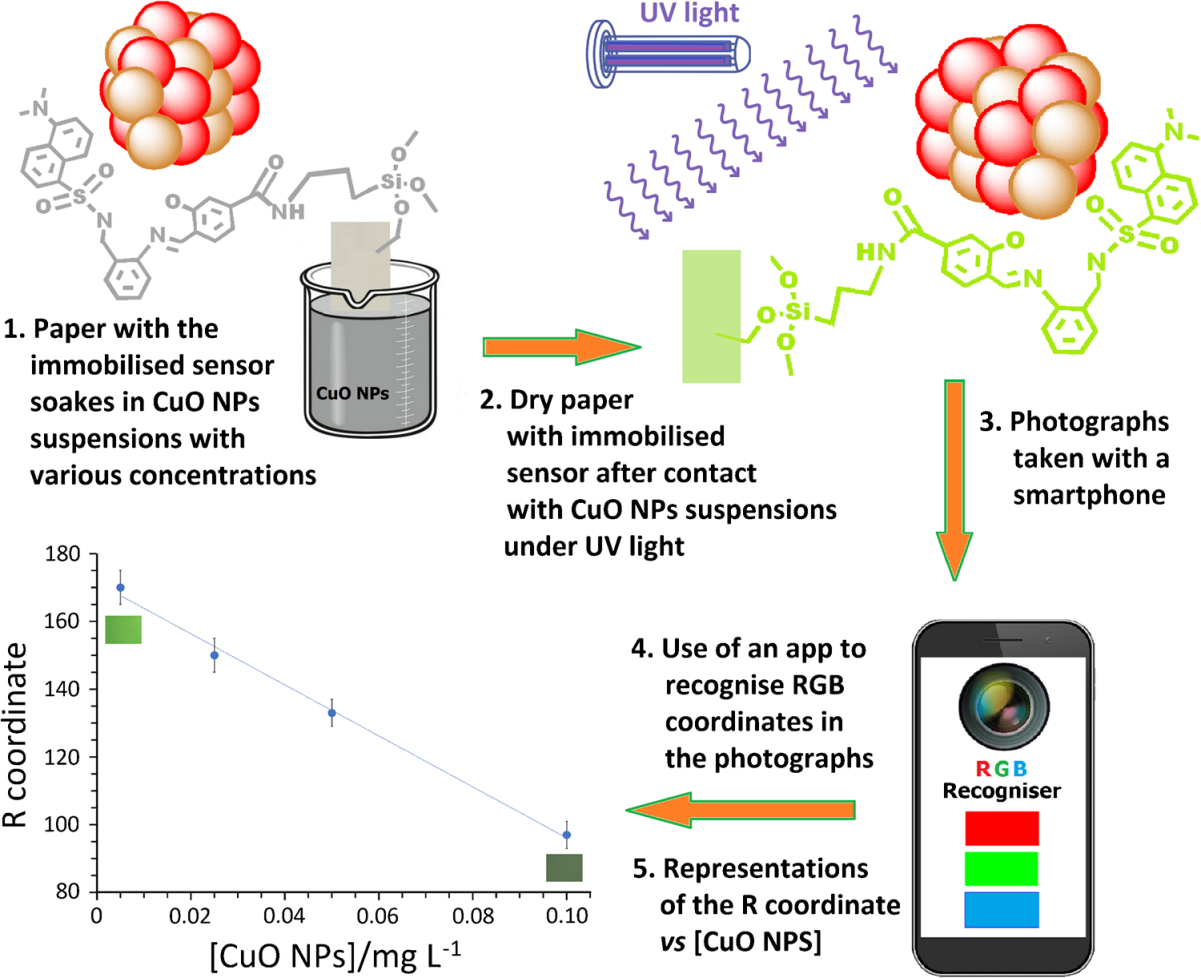
Schematic representation of the sensing strategy

Smartphone-based DIC is recognised as a powerful, rapid, and low-cost analytical approach to detect a wide range of analytes, including heavy metal ions, herbicides, pesticides, antibiotics, biochemical indicators, natural compounds, and bacteria/viruses [39, 53–55]. In contrast, there are few studies in which DIC is used for the detection of nanomaterials [51, 56]. Here, we present, to our knowledge, the first example of a colorimetric array approach for the rapid and sensitive identification of CuO NPs in aqueous media. A schematic illustration of the proposed sensing strategy is shown in Fig. 3.

After soaking for 15 min cellulose paper discs with immobilised chemosensor in water dispersions of CuO NPs with increasing concentrations from 5 to 100 µg L^−1^, these discs experimented, under natural light, a gradual darkening in the grey range, which is noticeable even to the naked eye. Similarly, a more evident darkening can be observed under an exciting UV light of 365 nm wavelength, as the colour of the paper changes from light green to a darker greyish green. This occurs as a result of the interaction between the CuO NPs and the chemosensor immobilised on the paper.

**Fig. 4:**
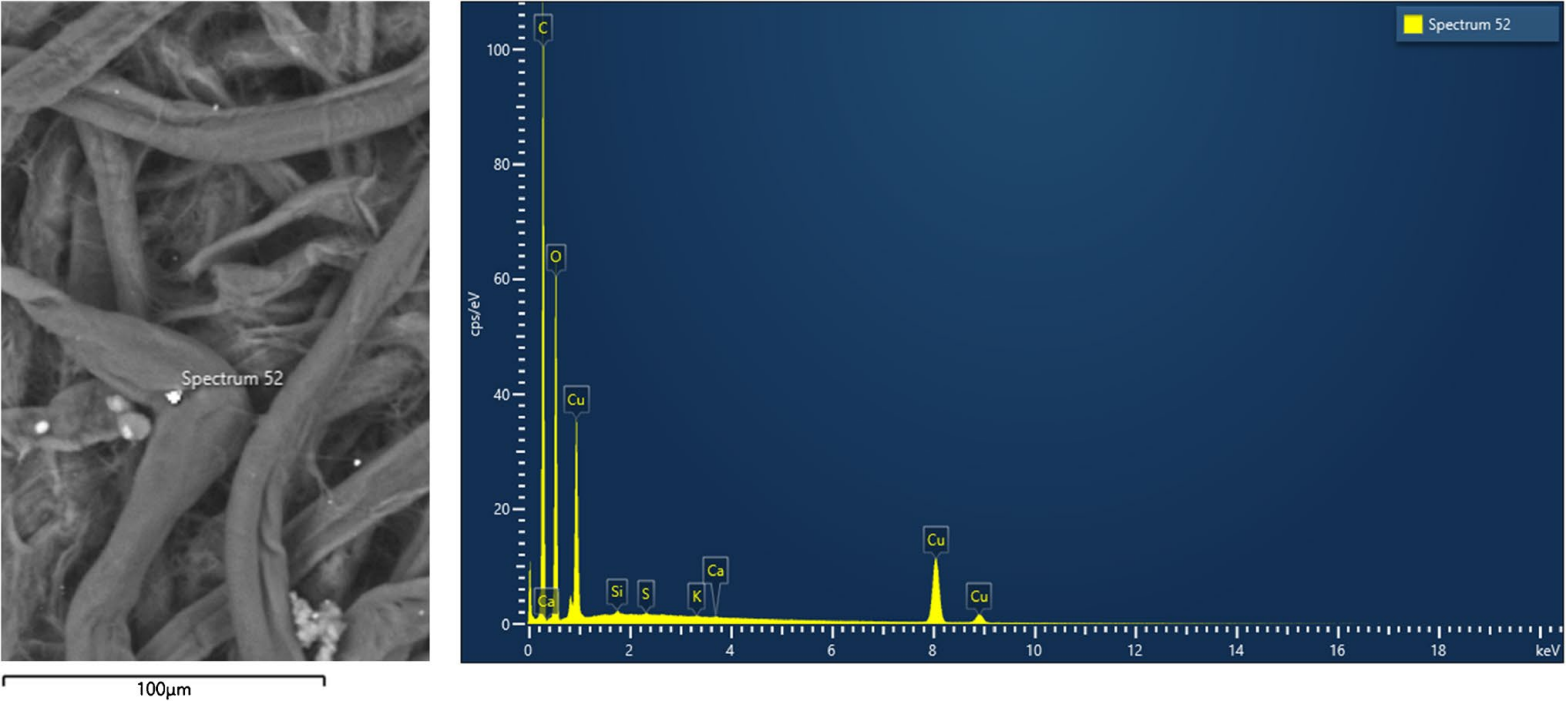
SEM image (left) and EDX spectrum (right) of the chemosensor cellulose paper after interaction with CuO NPs

SEM–EDX was employed to study the chemosensor cellulose paper after its interaction with CuO NPs. As shown in Fig. 4, crystalline CuO deposits were observed on the surface, indicating that the copper(II) species interacts with the chemosensor cellulose substrate. Copper presence was confirmed by the detection of its characteristic X-ray emission lines: Lα₁ at 0.931 keV, Kα₁ at 8.048 keV, and Kα₂ at 8.905 keV in the EDX spectrum of the chemosensor cellulose paper after its interaction with CuO NPs.

Based on DFT studies of the interaction between CuO NPs and the free chemosensor [50], we propose the formation of coordination bonds between surface Cu^2^⁺ ions and the azomethine, sulfonamide, and phenolic groups of the chemosensor.

To quantify these latter changes and facilitate their analysis, digitalised colour information was extracted from photographs obtained through an UV viewing cabinet under *λ*_ex_ = 365 nm light, with the built-in camera of an unsophisticated smartphone. Punctual data were transformed to RGB values employing a colour recogniser application implemented on the smartphone used for that purpose [39–41]. Colour information was obtained from three replicas of photographs taken on five points of different parts of each paper piece. The RGB coordinates were calculated through the free RGB Color Detector application [52].

**Fig. 5:**
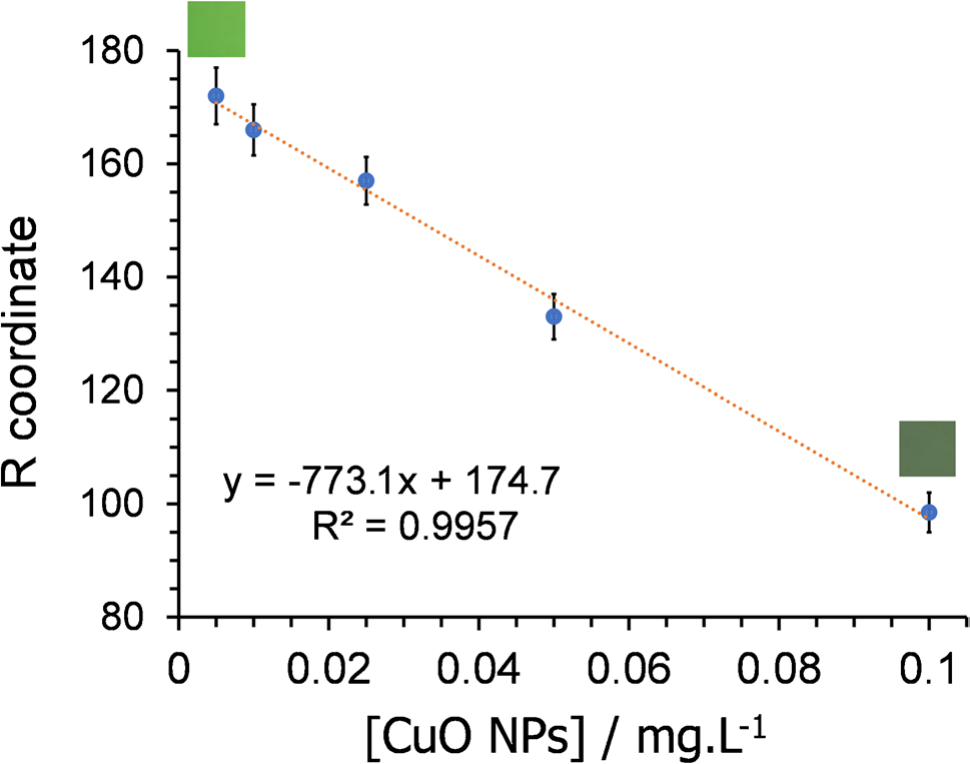
Variation of the red coordinate (R) in the RGB space, measured on photographs of the cellulose paper with the immobilised chemosensor under UV light (*λ*_ex_ = 365 nm), upon being soaked for 15 min in CuO NP water solutions with concentrations of 5, 10, 25, 50, and 100 µg L^−1^. The SD for the slope and the intercept were 37.6 and 2.2, respectively. Two images of the paper under UV light have been included to show the colour change

Since only the red coordinate (R) exhibited a clear sensitivity to CuO NPs, the response was only considered for this channel, as an average of the values measured. The variation of the blue (B) and green (G) coordinates with the concentration of CuO NPs is not lineal and therefore does not deserve further attention. Figure 5 shows a satisfactory linear correlation between this R-coordinate and the CuO NP concentration, being *R*^2^ = 0.9957. The slope average of three different calibration lines was −773.1, with a standard deviation of 37.6.

Sensitivity was evaluated through LOD and LOQ, using 10 replicas of blanks. The values LOD and LOQ of this chemosensor could be extracted from LOD = 3SD/M and LOQ = 10SD/M, correspondingly, being SD the standard deviation of the response, while *M* represents the slope shown by the calibration curve [57]. These values were estimated from the slope of the calibration curve (–773.1) and the standard deviation of the sample without CuO NPs (2.7). Hence, CuO NPs could be detected with a LOD of 10.5 µg L^−1^ and a LOQ of 34.9 µg L^−1^, indicating an even higher sensitivity than that reported by us for another chemosensor in solution (LOD = 13.83 µg L^−1^ and LOQ = 46.05 µg L^−^^1^) [37].

The precision of the colorimetric method was assessed through an interday study conducted over three non-consecutive days. On each day, a calibration curve was constructed within the concentration range of 5–100 µg·L⁻^1^ of CuO NPs. Furthermore, 15, 50, and 100 µg·L⁻^1^ CuO NP suspensions were freshly prepared each day and measured in triplicate. The precision was calculated using the relative standard deviation (RSD = 100 *S*/*x*, being *S* the standard deviation, while *x* represents the mean of the data) at three concentration levels within the linear range (15, 50, and 100 µg L^−1^). The obtained values of RSD, 3%, 1%, and 1% for the concentration levels set (15, 50, and 100 µg L^−1^) are indicative of excellent precision in the reproducibility of the results.

Analytical recovery (AR) was assessed using tap water samples spiked with 15, 50, and 100 µg·L⁻^1^ of CuO NPs over three non-consecutive days. The recovery percentage was calculated using the following equation: AR = 100 FC/AC, where FC stands for the found concentration of CuO NPs, and AC means the concentration of CuO NPs added. The mean recoveries obtained, 109 ± 9%, 102 ± 1%, and 99 ± 1% for the concentration levels set (15, 50 µg L^−1^ and 100 µg L^−1^, respectively), reveal the accuracy of these measurements.

### DR for CuO NP detection

We have also investigated the use of UV/Vis/NIR spectrophotometry to monitor those colour changes undergone by the chemosensor-cellulose paper upon immersion in a CuO NP suspension. Before conducting the study, the immersion time of the chemosensor paper was investigated.

#### Time of immersion for DR

A study was carried out to determine the immersion time required for absorbance to stabilise when cellulose paper is exposed to a dispersion of CuO NPs. For this purpose, six paper samples were immersed in an aqueous suspension of CuO NPs at a concentration of 20 mg·L⁻^1^. The evaluated immersion times were 15, 30, 45, 60, and 120 min, as well as overnight. After each interval, the samples were removed from the suspension and allowed to air dry. Visual inspection showed that prolonged exposure to CuO NPs led to a progressively darker coloration of the paper. Absorbance was measured using DR spectroscopy. Figure S5 (SI) shows that the area under the absorption peak at 270 nm decreased with increasing immersion time. However, after 2 h, the signal stabilised, with no significant difference compared to the sample immersed overnight.

#### DR colorimetric detection of CuO NPs

**Fig. 6:**
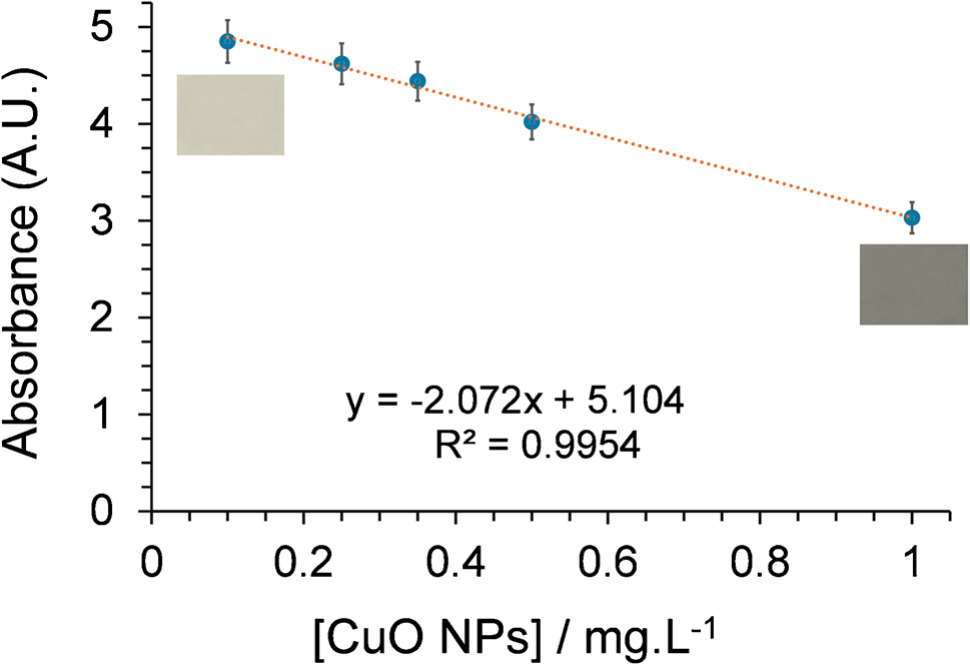
Absorbance of the characteristic band centred at 270 nm of the chemosensor immobilised on cellulose paper, with increasing concentrations of CuO NPs (100, 250, 350, 500, and 1000 µg·L⁻^1^. The SD for the slope and the intercept were 0.091 and 0.054, respectively. Two small images of the paper have been included to show the colour change experimented

Chemosensor-modified cellulose paper discs were soaked for 2 h in CuO NP water suspensions, whose concentration increased from 100 to 1000 µg L^−1^. As a result, the discs underwent a gradual darkening within the grey range, which is visible to the naked eye. The absorbance of the DR spectrum of the dry papers exhibited a gradual decrease for all the bands. However, since the band at 270 nm was that showing the most significant decrease in the absorbance, we decided to use these values to correlate the response of the immobilised chemosensor to the CuO NP concentration. The satisfactory linear decrease in this absorbance band is shown in Fig. 6, where the CuO NP concentration increases from 100 to 1000 µg L^−1^. The results of LOD and LOQ calculations were 100.0 and 333.4 µg L^−1^, respectively. These results indicate a good sensitivity, although less satisfactory than for the use of the colour recogniser app implemented on the smartphone. A direct comparison using identical CuO NP samples and chemosensor papers was not feasible, as the linear response ranges of the red coordinate and absorbance do not overlap due to differences in sensitivity.

The precision of the DR method was assessed through an interday study conducted over three non-consecutive days. On each day, a calibration curve was constructed within the concentration range of 100 to 1000 μg·L⁻^1^ of CuO NPs. The obtained values of RSD (ca. 4%, 8%, and 3%, for the set concentration levels of 350, 500, and 1000 µg L^−1^, respectively) also disclose a good precision in the reproducibility of the results, but again, not as good as that obtained using the colour recogniser app. Similarly, the obtained AR values of 92 ± 4%, 109 ± 8%, and 98 ± 3% for the set concentration levels (350, 500, and 1000 µg L^−1^, respectively) demonstrate an acceptable accuracy of the measurements.

### Fluorescence spectroscopy for CuO NP detection

Fluorescence quenching experiments were also conducted to evaluate the effectiveness of the dansyl-based chemosensor for detecting CuO NPs dissolved in an ethanol:water mixture (80:20). Drawing on our previous experience with this chemosensor in solution [50], we selected a concentration of 1.26 µg L^−1^ that resulted in an approximate 90% decrease in fluorescence emission intensity, upon interaction with NP concentrations in the ppb range. Since a previous study of the influence of pH on the fluorescence spectrum of the chemosensor showed an increase of 2.5 times in fluorescence intensity at pH = 9 in relation to near-neutrality pH, this study was performed at pH 9. This facilitated a stronger interaction between the CuO NPs and the chemosensor, further enhancing the stability of the suspension. An excitation wavelength of 400 nm was used, as it resulted in the highest fluorescence emission of the chemosensor (see “Fluorescence and colorimetric characterisation of the chemosensor” section for details).

#### Fluorometric detection of CuO NPs

**Fig. 7:**
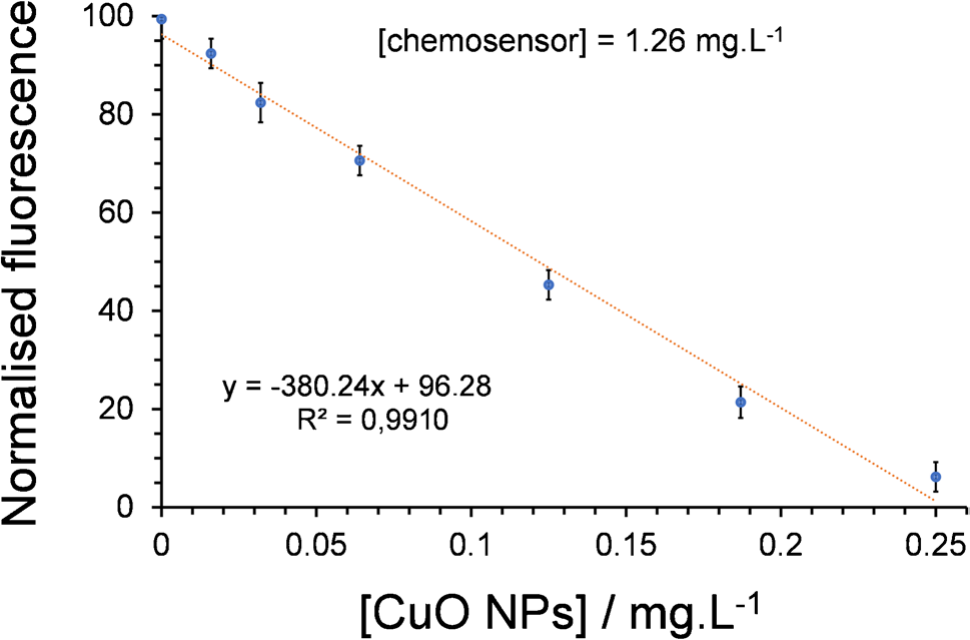
Calibration curve of the fluorescence emission spectrum of the free chemosensor dissolved in an EtOH:H_2_O (80:20) solution (pH = 9), with increasing concentrations of CuO NPs (16, 32, 64, 125, 187, and 250 µg·L⁻^1^). The SD of the slope and the intercept were 16.20 and 2.11, respectively

Fluorescence quenching experiments shown the effectiveness of the dansyl-based chemosensor for detecting CuO NPs dispersed in an ethanol:water mixture (80:20). Figure 7 shows a linear decrease of over 90% in the fluorescence intensity (*λ*_em_ = 520 nm) of the free chemosensor (1.26 µg·L⁻^1^) in an ethanol–water (80:20, v/v) solution upon the addition of CuO NPs at concentrations ranging from 0 to 250 µg·L⁻^1^.

Calculated LOD and LOQ for CuO NPs, with values of 30 and 100 µg L^−1^, respectively, are a sign of good sensitivity [37]. Using a chemosensor concentration of only 1.26 µg L^−1^, the working range for detecting CuO NPs was 100–250 µg L^−1^. The working range of this chemosensor was deduced from the minimum measurable value (LOQ) and the highest concentration of CuO NPs that maintains linearity.

### Selectivity of the dissolved chemosensor toward CuO NP detection

**Fig. 8:**
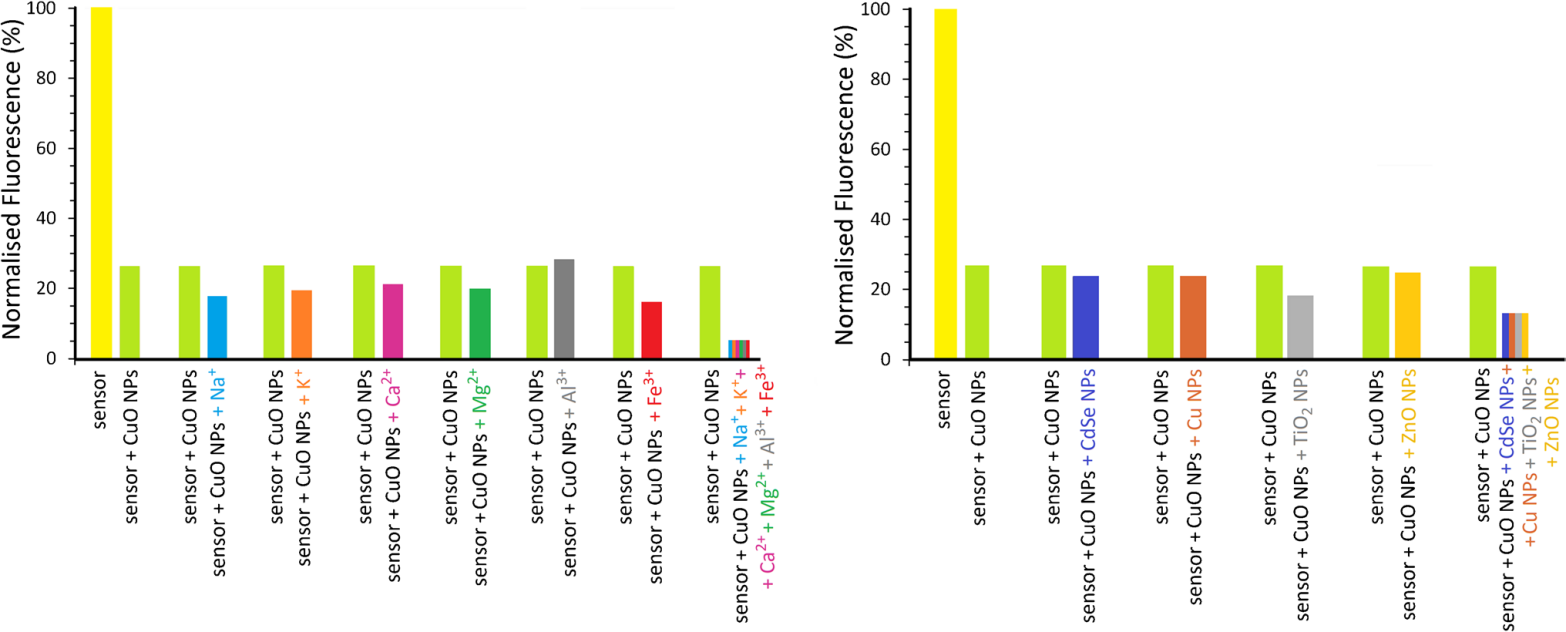
Right: Bar chart showing the fluorescence intensity of the chemosensor (1.26 µg L⁻^1^) in response to CuO NPs (80 µg L⁻^1^), with the addition of different NPs as potential interferents. CdSe and Cu NPs were tested at the same concentration as CuO (80 µg L⁻^1^), whereas TiO₂ and ZnO NPs were tested at 40 µg L⁻^1^. Left: Interference study with Na⁺, K⁺, Fe^3^⁺, Mg^2^⁺, Ca^2^⁺ (80 µg L⁻^1^), and Al^3^⁺ (40 µg L⁻^1^). Measurements were carried out in ethanol–water mixtures (80:20, v/v) adjusted to pH 9, with excitation at 400 nm

The selectivity of the free dansyl-based chemosensor toward CuO NPs was systematically assessed in 80:20 ethanol–water mixtures, in the presence of representative metal ions (K⁺, Na⁺, Mg^2^⁺, Ca^2^⁺, Al^3^⁺, and Fe^3^⁺) and common engineered NPs such as those composed of Cu, CdSe, ZnO, and TiO₂ (Fig. 8). The concentrations of the dissolved chemosensor and CuO NPs were fixed at 1.26 µg L⁻^1^ and 80 µg L⁻^1^, respectively. Each assay was performed in triplicate, and a deviation of ± 10% from the average fluorescence intensity was used as a criterion to determine potential interference.

As shown in Fig. 8, the presence of Cu and CdSe nanoparticles, as well as Na⁺, K⁺, Mg^2^⁺, Ca^2^⁺, and Fe^3^⁺ ions at 80 µg L⁻^1^, did not significantly affect the fluorescence response of the sensor toward CuO NPs, indicating negligible interference under these conditions. In contrast, Al^3^⁺ ions and ZnO and TiO₂ nanoparticles showed some influence at higher concentrations; however, their interference remained within acceptable limits when their concentrations were reduced to 40 µg L⁻^1^ or below. These results demonstrate that the chemosensor exhibits good selectivity toward CuO NPs, even in the presence of potentially competing species. Nevertheless, in complex matrices (as illustrated by the bar representing the combined presence of species in each panel of Fig. 8), there is a risk of false positive signals caused by competing species, particularly metal ions, at the sensor’s recognition sites. Therefore, this issue should be carefully considered and addressed in future studies.

## Conclusions

The main innovative contributions that set our approach apart from previous studies and underscore the practical relevance and originality of the proposed sensing platform are summarised as follows:

i) Development of a dual-mode chemosensor that integrates both colorimetric and fluorescence detection, significantly enhancing sensitivity and reliability in analyte recognition.
ii) Incorporation of smartphone-based quantification and image analysis, enabling a portable, user-friendly, and on-site detection platform that operates without the need for sophisticated instrumentation
iii) Adoption of a simple, low-cost fabrication process that is not only accessible but also amenable to large-scale production and practical use in field conditions.

We have shown the usefulness of smartphone-based DIC for detecting CuO NPs with a chemosensor covalently immobilised on cellulose paper. Calculated LOD (10.5 µg L^−1^) and LOQ (34.9 µg L^−1^) showed the excellent sensitivity of this simple method. The values of RSD of 1% and AR of 99 ± 1% at 100 µg L^−1^ are indicative of good precision in the reproducibility of the results and in the accuracy of the measurement, respectively.

DR spectrometry also allowed to discern the colour changes displayed by the dansyl chemosensor immobilised on cellulose paper, with acceptable parameters for the detection of CuO NPs. However, it does not reach the levels of sensitivity (LOD = 100.0 µg L^−1^ and LOQ = 333.4 µg L^−1^), precision (RSD = 3% at 1000 µg L^−1^), and accuracy (AR = 98 ± 3 at 1000 µg L^−1^) obtained with the smartphone colour recognition app.

In solution, the free chemosensor showed a linear fluorescence-quenching response to CuO NPs in the range 0–250 µg L^−1^. Calculated LOD (30 µg L^−1^) and LOQ (100 µg L^−1^) indicate a good sensitivity of the method. Using a chemosensor concentration of only 1.26 µg L^−1^, the working range for detecting CuO NPs was 100–250 µg L^−1^. Likewise, the dissolved chemosensor displayed a satisfactory selectivity towards CuO NPs in the presence of several metal ions habitual in water, as well as other common nanomaterials as Cu NPs and CdSe NPs being present. Thus, concentrations of 80 µg L^−1^ of these latter ones or for K^+^, Na^+^, Ca^2+^, Mg^2+^, and Fe^3+^ ions do not interfere significatively to determine concentrations of CuO NPs. In the case of Al^3+^ ions and other nanomaterials as ZnO NPs or TiO_2_ NPs, they can be tolerated without interferences in concentrations of 40 µg L^−1^ without interference.

In view of the fast response, sensitivity, precision, and accuracy obtained by DIC using a simple smartphone, this method seems a useful tool to be considered in the detection of CuO NPs. It must be noted that this study has been conceived as an initial step in a broader investigation focused on applying DIC to the quantification of nanomaterials in real samples. Since real water samples contain various organic and inorganic substances, suspended solids, and biological matter, it is to be expected that they may interfere with the analytical process and affect the accuracy and precision of the results. Further research on the topic remains necessary to anticipate and address these and other predictable challenges. This simple system, tested with lab-prepared samples, may serve as a basis for future studies on the subject.

## Supplementary Information

Below is the link to the electronic supplementary material.

ESM 1 DOCX (1.10 MB)

## Data Availability

No datasets were generated or analysed during the current study.

## References

[1] Gnanavel V, Palanichamy V, Mohana Roopan S (2017) Biosynthesis and characterization of copper oxide nanoparticles and its anticancer activity on human colon cancer cell lines (HCT-116). J Photochem Photobiol B 171:133–138. 10.1016/j.jphotobiol.2017.05.001 28501691 10.1016/j.jphotobiol.2017.05.001

[2] Ishaque Nabila M, Kannabiran K (2018) Biosynthesis, characterization and antibacterial activity of copper oxide nanoparticles (CuO NPs) from actinomycetes. Biocatal Agric Biotechnol 15:56. 10.1016/j.bcab.2018.05.011

[3] Jadhav MS, Kulkarni S, Raikar P, Barretto DA, Vootla SK, Raikar US (2018) Green biosynthesis of CuO & Ag–CuO nanoparticles from *Malus domestica* leaf extract and evaluation of antibacterial, antioxidant and DNA cleavage activities. New J Chem 42:204–213. 10.1039/C7NJ02977B

[4] Ringu T, Das A, Ghosh S, Pramanik N (2024) Exploring the potential of copper oxide nanoparticles (CuO NPs) for sustainable environmental bioengineering applications. Nanotechnol Environ Eng 9:679–707. 10.1007/s41204-024-00389

[5] Saleem MH, Ejaz U, Vithanage M, Bolan N, Siddique KHM (2024) Synthesis, characterization, and advanced sustainable applications of copper oxide nanoparticles: a review. Clean Technol Environ. 10.1007/s10098-024-02774-6

[6] Jayasimha HN, Chandrappa KG, Sanaulla PF, Dileepkumar VG (2024) Green synthesis of CuO nanoparticles: a promising material for photocatalysis and electrochemical sensor. Sens Int 5:100254. 10.1016/j.sintl.2023.100254

[7] Mallakpour S, Azadi E, Hussain CM (2020) Environmentally benign production of cupric oxide nanoparticles and various utilizations of their polymeric hybrids in different technologies. Coord Chem Rev 419:213378. 10.1016/j.ccr.2020.213378

[8] Kana N, Kaviyarasu K, Khamliche T, Magdalane CM, Maaza M (2019) Stability and thermal conductivity of CuO nanowire for catalytic applications. J Environ Chem Eng 7:103255. 10.1016/j.jece.2019.103255

[9] Ighalo JO, Sagboye PA, Umenweke G, Ajala OJ, Omoarukhe FO, Adeyanju CA, Ogunniyi S, Adeniyi AG (2021) CuO nanoparticles (CuO NPs) for water treatment: a review of recent advances. Environ Nanotechnol Monit Manag 15:100443. 10.1016/j.enmm.2021.100443

[10] Sibhatu AK, Weldegebrieal GK, Sagadevan S, Tran NN, Hessel V (2022) Photocatalytic activity of CuO nanoparticles for organic and inorganic pollutants removal in wastewater remediation. Chemosphere 300:134623. 10.1016/j.chemosphere.2022.134623 35439489 10.1016/j.chemosphere.2022.134623

[11] Mishra SR, Ahmaruzzaman M (2022) CuO and CuO-based nanocomposites: synthesis and applications in environment and energy. Sustain Mater Techno 33:e00463. 10.1016/j.susmat.2022.e00463

[12] Chauhan M, Sharma B, Kumar R, Chaudhary GR, Hassan AA, Kumar S (2019) Green synthesis of CuO nanomaterials and their proficient use for organic waste removal and antimicrobial application. Environ Res 168:85–95. 10.1016/j.envres.2018.09.024 30278366 10.1016/j.envres.2018.09.024

[13] Akintelu SA, Folorunso AS, Folorunso FA, Oyebamiji AK (2020) Green synthesis of copper oxide nanoparticles for biomedical application and environmental remediation. Heliyon 6:e04508. 10.1016/j.heliyon.2020.e04508 32715145 10.1016/j.heliyon.2020.e04508 PMC7378697

[14] Wang Z, Li N, Zhao J, White Jc, Qu P, Xing B (2012) CuO nanoparticle interaction with human epithelial cells: cellular uptake, location, export, and genotoxicity. Chem Res Toxicol 25:1512–1521. 10.1021/tx3002093 22686560 10.1021/tx3002093

[15] Ahamed M, Akhtar MJ, Alhadlaq HA, Alrokayan SA (2015) Assessment of the lung toxicity of copper oxide nanoparticles: current status. Nanomedicine 10:2365–2377. 10.2217/nnm.15.72 26251192 10.2217/nnm.15.72

[16] Akhtar MJ, Kumar S, Alhadlaq HA, Alrokayan SA, Abu-Salah KM, Ahamed M (2016) Dose-dependent genotoxicity of copper oxide nanoparticles stimulated by reactive oxygen species in human lung epithelial cells. Toxicol Ind Health 32:809–821. 10.1177/0748233713511512 24311626 10.1177/0748233713511512

[17] Pourahmad J, Salami M, Zarei MH (2023) Comparative toxic effect of bulk copper oxide (CuO) and CuO nanoparticles on human red blood cells. Biol Trace Elem Res 201:149–155. 10.1007/s12011-022-03149-y 35378668 10.1007/s12011-022-03149-y

[18] Ameh T, Sayes CM (2019) The potential exposure and hazards of copper nanoparticles: a review. Environ Toxicol Pharmacol 71:103220. 10.1016/j.etap.2019.103220 31306862 10.1016/j.etap.2019.103220

[19] Naz S, Gul A, Zia M (2020) Toxicity of copper oxide nanoparticles: a review study. IET Nanobiotechnol 14:1–13. 10.1049/iet-nbt.2019.0176 31935671 10.1049/iet-nbt.2019.0176 PMC8676634

[20] Rajput V, Minkina T, Sushkova S, Behal A, Maksimov A, Blicharska E, Ghazaryan K, Movsesyan H, Barsova N (2020) *ZnO* and *CuO* nanoparticles: a threat to soil organisms, plants, and human health. Environ Geochem Health 42:147–158. 10.1007/s10653-019-00317-3 31111333 10.1007/s10653-019-00317-3

[21] Hou J, Wang X, Hayat T, Wang X (2017) Ecotoxicological effects and mechanism of CuO nanoparticles to individual organisms. Environ Pollut 221:209–217. 10.1016/j.envpol.2016.11.066 27939631 10.1016/j.envpol.2016.11.066

[22] Du J, Fu L, Li H, Xu S, Zhou Q, Tang J (2019) The potential hazards and ecotoxicity of CuO nanoparticles: an overview. Toxin Rev 40:460–472. 10.1080/15569543.2019.1670211

[23] Moschini E, Colombo G, Chirico G, Capitani G, Dalle-Donne I, Mantecca P (2023) Biological mechanism of cell oxidative stress and death during short-term exposure to nano CuO. Sci Rep 13:2326. 10.1038/s41598-023-28958-6 36759527 10.1038/s41598-023-28958-6 PMC9911756

[24] Sajjad H, Sajjad A, Haya RT, Khan MM, Zia M (2023) Copper oxide nanoparticles: in vitro and in vivo toxicity, mechanisms of action and factors influencing their toxicology. Comp Biochem Physiol C Toxicol Pharmacol 271:109682. 10.1016/j.cbpc.2023.109682 37328134 10.1016/j.cbpc.2023.109682

[25] Murugesan S, Balasubramanian S, Perumal E (2025) Copper oxide nanoparticles induced reactive oxygen species generation: a systematic review and meta-analysis. Chem Biol Interact 405:111311. 10.1016/j.cbi.2024.111311 39551423 10.1016/j.cbi.2024.111311

[26] Assadian E, Zarei MH, Gilani AG, Farshin M, Degampanah H, Pourahmad J (2018) Toxicity of copper oxide (CuO) nanoparticles on human blood lymphocytes. Biol Trace Elem Res 184:350–357. 10.1007/s12011-017-1170-4 29064010 10.1007/s12011-017-1170-4

[27] Navratilova J, Praetorius A, Gondikas A, Fabienke W, Kammer FVD, Hofmann T (2015) Detection of engineered copper nanoparticles in soil using single particle ICP-MS. Int J Environ Res Public Health 12:15756–15768. 10.3390/ijerph121215020 26690460 10.3390/ijerph121215020 PMC4690956

[28] Laughton S, Laycock A, Bland G, von der Kammer F, Hofmann T, Casman EA, Lowry GV (2021) Methanol-based extraction protocol for insoluble and moderately water-soluble nanoparticles in plants to enable characterization by single particle ICP-MS. Anal Bioanal Chem 413:299–314 33123761 10.1007/s00216-020-03014-8

[29] Laughton S, Laycock A, von der Kammer F, Hofmann T, Casman EA, Rodrigues SM, Lowryet GV (2019) Persistence of copper-based nanoparticle-containing foliar sprays in Lactuca sativa (lettuce) characterized by spICP-MS. J Nanopart Res 21:174. https://doi-org.ezbusc.usc.gal/10.1007/s11051-019-4620-4.

[30] Bland GD, Lowry GV (2020) Multistep method to extract moderately soluble copper oxide nanoparticles from soil for quantification and characterization. Anal Chem 92:9620–9628 32520530 10.1021/acs.analchem.0c00824

[31] Majedi SM, Kelly BC, Lee HK (2014) Evaluation of a cloud point extraction approach for the preconcentration and quantification of trace CuO nanoparticles in environmental waters. Anal Chim Acta 814:39–48. 10.1016/j.aca.2014.01.022 24528842 10.1016/j.aca.2014.01.022

[32] Zhou XX, Liu JF, Geng FL (2016) Determination of metal oxide nanoparticles and their ionic counterparts in environmental waters by size exclusion chromatography coupled to ICP-MS. NanoImpact 1:13–20. 10.1016/j.impact.2016.02.002

[33] Peng C, Xu C, Liu Q, Sun L, Luo Y, Shi J (2017) Fate and transformation of CuO nanoparticles in the soil–rice system during the life cycle of rice plants. Environ Sci Technol 51:4907–4917. 10.1021/acs.est.6b05882 28383251 10.1021/acs.est.6b05882

[34] Zhao J, Ren W, Dai Y, Liu L, Wang Z, Yu X, Zhang J, Wang X, Xing B (2017) Uptake, distribution, and transformation of CuO NPs in a floating plant *Eichhornia crassipes* and related stomatal responses. Environ Sci Technol 51:7686–7695. 10.1021/acs.est.7b01602 28586199 10.1021/acs.est.7b01602

[35] Mortimer M, Gogos A, Bartolomé N, Kahru A, Bucheli TD, Slaveykova VI (2014) Potential of hyperspectral imaging microscopy for semi-quantitative analysis of nanoparticle uptake by protozoa. Environ Sci Technol 48:8760–8767. 10.1021/es500898j 25000358 10.1021/es500898j

[36] Vasco MS, Alves LC, Corregidor V, Correia D, Godinho CP, Sá-Correia I, Bettiol A, Watt F, Pinheiro T (2017) 3D map distribution of metallic nanoparticles in whole cells using MeV ion microscopy. J Microsc 267:227–236 28394445 10.1111/jmi.12561

[37] Sanmartín-Matalobos J, García-Deibe AM, Fondo M, Zarepour-Jevinani M, Domínguez-González MR, Bermejo-Barrera P (2017) Exploration of an easily synthesized fluorescent probe for detecting copper in aqueous samples. Dalton Trans 46:15827–15835. 10.1039/C7DT02872E 29109993 10.1039/c7dt02872e

[38] Sanmartín-Matalobos J, García-Deibe AM, Zarepour-Jevinani M, Aboal-Somoza M, Bermejo-Barrera P, Fondo M (2020) Exploring the chelating potential of an easily synthesized Schiff base for copper sensing. Crystals 10:235. 10.3390/cryst10030235

[39] Fan Y, Li J, Guo Y, Xie L, Zhang G (2021) Digital image colorimetry on smartphone for chemical analysis: a review. Measurement 171:108829. 10.1016/j.measurement.2020.108829

[40] Yang F, Lin D, Pan L, Zhu J, Shen J, Yang L, Jiang C (2021) Portable smartphone platform based on a single dual-emissive ratiometric fluorescent probe for visual detection of isopropanol in exhaled breath. Anal Chem 93:14506–14513. 10.1021/acs.analchem.1c03280 34609831 10.1021/acs.analchem.1c03280

[41] Yan F, Hu S, Wang Y, Song X, Cao C, Wang K, Jing C, Zhang G, Liu W (2021) A multifunctional fluorescent probe for visualizing H_2_S in wastewater with portable smartphone via fluorescent paper strip and sensing GSH *in vivo*. J Hazard Mater 406:124523. 10.1016/j.jhazmat.2020.124523 33310319 10.1016/j.jhazmat.2020.124523

[42] Guo L, Liu H, Peng F, Qi H (2021) Efficient and portable cellulose-based colorimetric test paper for metal ion detection. Carbohydr Polym 274:118635. 10.1016/j.carbpol.2021.118635 34702458 10.1016/j.carbpol.2021.118635

[43] Jiang X, Xia J, Luo X (2020) Simple, rapid, and highly sensitive colorimetric sensor strips from a porous cellulose membrane stained with victoria blue B for efficient detection of trace Cd(II) in water. ACS Sustain Chem Eng 8:5184–5191. 10.1021/acssuschemeng.9b07614

[44] Zhang X, Huang J (2010) Functional surface modification of natural cellulose substances for colorimetric detection and adsorption of Hg^2+^ in aqueous media. Chem Commun 46:6042–6044. 10.1039/C0CC01072C 10.1039/c0cc01072c 20514381

[45] Chen W, Fang X, Li H, Cao H, Kong J (2016) A simple paper-based colorimetric device for rapid mercury(II) assay. Sci Rep 6:31948. 10.1038/srep31948 27554633 10.1038/srep31948 PMC4995402

[46] Li M, Li X, Xiao HN, James TD (2017) Fluorescence sensing with cellulose-based materials. ChemistryOpen 6(6):685–696. 10.1002/open.201700133 29226055 10.1002/open.201700133 PMC5715359

[47] Nawaz H, Chen S, Li X, Zhang X, Zhang X, Wang JQ, Xu F (2022) Cellulose-based environment-friendly smart materials for colorimetric and fluorescent detection of Cu^2+^/Fe^3+^ ions and their anti-counterfeiting applications. Chem Eng J 438:135595. 10.1016/j.cej.2022.135595

[48] Gabrielli V, Frasconi M (2022) Cellulose-based functional materials for sensing. Chemosensors 10:352. 10.3390/chemosensors10090352

[49] Qiu C, Liu H, Wang X, Tao S, Mo J, Chen P, Xiao H, Qi H (2024) Cellulose-based fluorescent chemosensor with controllable sensitivity for Fe^3+^ detection. Carbohydr Polym 346:122620. 10.1016/j.carbpol.2024.122620 39245528 10.1016/j.carbpol.2024.122620

[50] Sanmartín-Matalobos J, Bermejo-Barrera P, Pérez-Juste I, Fondo M, García-Deibe AM, Alves-Iglesias Y (2022) Experimental and computational studies on the interaction of a dansyl-based fluorescent Schiff base ligand with Cu^2+^ ions and CuO NPs. Int J Mol Sci 23:11565. 10.3390/ijms231911565 36232868 10.3390/ijms231911565 PMC9569476

[51] Sanmartín-Matalobos J, Bermejo-Barrera P, Pérez-Juste I, Fondo M, García-Deibe AM, Alves-Iglesias Y (2022) Detecting CdSe nanomaterials with a fluorescent Schiff base ligand. Chemosensors 10:394. 10.3390/chemosensors10100394 10.3390/ijms231911565 PMC9569476 36232868

[52] RGB Color Detector, version 201.0.0 by The Programmer (2025) https://play.google.com/store/apps/details?id=com.TheProgrammer.RGBColorDetector&hl=en-GB. Accessed 5 Aug 2025

[53] Markus V, Paul AA, Marks RS, Caleb J (2025) Smartphone digital image colorimetry: an affordable easy-to-use technique. Anal Lett 1–19:1. 10.1080/00032719.2025.2474573

[54] Choodum A, Sriprom W, Wongniramaikul W (2019) Portable and selective colorimetric film and digital image colorimetry for detection of iron. Spectrochim Acta A Mol Biomol Spectrosc 208:40–47. 10.1016/j.saa.2018.09.062 30292149 10.1016/j.saa.2018.09.062

[55] Shabrina AN, Nursa’adah E, Firdaus ML (2025) Quantitative detection of hexavalent chromium in aquatic environments using digital image colorimetry and the green chemistry approach. ACS Sustainable Chem Eng. 10.1021/acssuschemeng.5c02305

[56] Mahmoudi M, Lohse SE, Murphy CJ, Suslick KS (2016) Identification of nanoparticles with a colorimetric sensor array. ACS Sens 1:17–21. 10.1021/acssensors.5b00014

[57] Shrivastava A, Gupta V (2011) Methods for the determination of limit of detection and limit of quantitation of the analytical methods. Chron Young Sci 2(1):21. 10.4103/2229-5186.79345

