# Supplementary material for: Convenient colorimetric approach to quantify CuO NPs in water using only a smartphone and cellulose paper with an immobilised chemosensor
Source: Mikrochim Acta. 2025 Aug 16;192(9):594. doi: 10.1007/s00604-025-07439-9 (PMC12356722; doi:10.1007/s00604-025-07439-9)
Supplement: Supplementary file 1 — DOCX (1.10 MB) [file 604_2025_7439_MOESM1_ESM.docx]

**Supplementary Information**

**Microchimica Acta**

**Convenient colorimetric approach to quantify CuO NPs in water using only a smartphone, and cellulose paper with an immobilised chemosensor**

Jesús Sanmartín-Matalobos^1,2,*^, Pilar Bermejo-Barrera^1,3^, Ana M. García-Deibe^2^, Matilde Fondo^2^, Yeneva Alves-Iglesias^2,3^

^1^ Institute of Materials (iMATUS), Avenida do Mestre Mateo 25, Universidade de Santiago de Compostela, 15782 Santiago de Compostela, Spain

^2^ Coordination and Supramolecular Chemistry Group (SupraMetal), Department of Inorganic Chemistry, Faculty of Chemistry, Avnda. das Ciencias s/n. Universidade de Santiago de Compostela, Avda. das Ciencias s/n, Campus Vida, 15782. Santiago de Compostela, Spain

^3^ Trace Element, Speciation and Spectroscopy Group (GETEE), Department of Analytical Chemistry, Nutrition and Bromatology, Faculty of Chemistry, Universidade de Santiago de Compostela, Campus Vida. 15782. Santiago de Compostela, Spain

*Corresponding author: [jesus.sanmartin@usc.es](mailto:jesus.sanmartin@usc.es)

*Supporting Characterization Data for Sections 3.1–3.5 of the Main Text*

*3.1* *Immobilising of the chemosensor on cellulose paper. Spectral data*


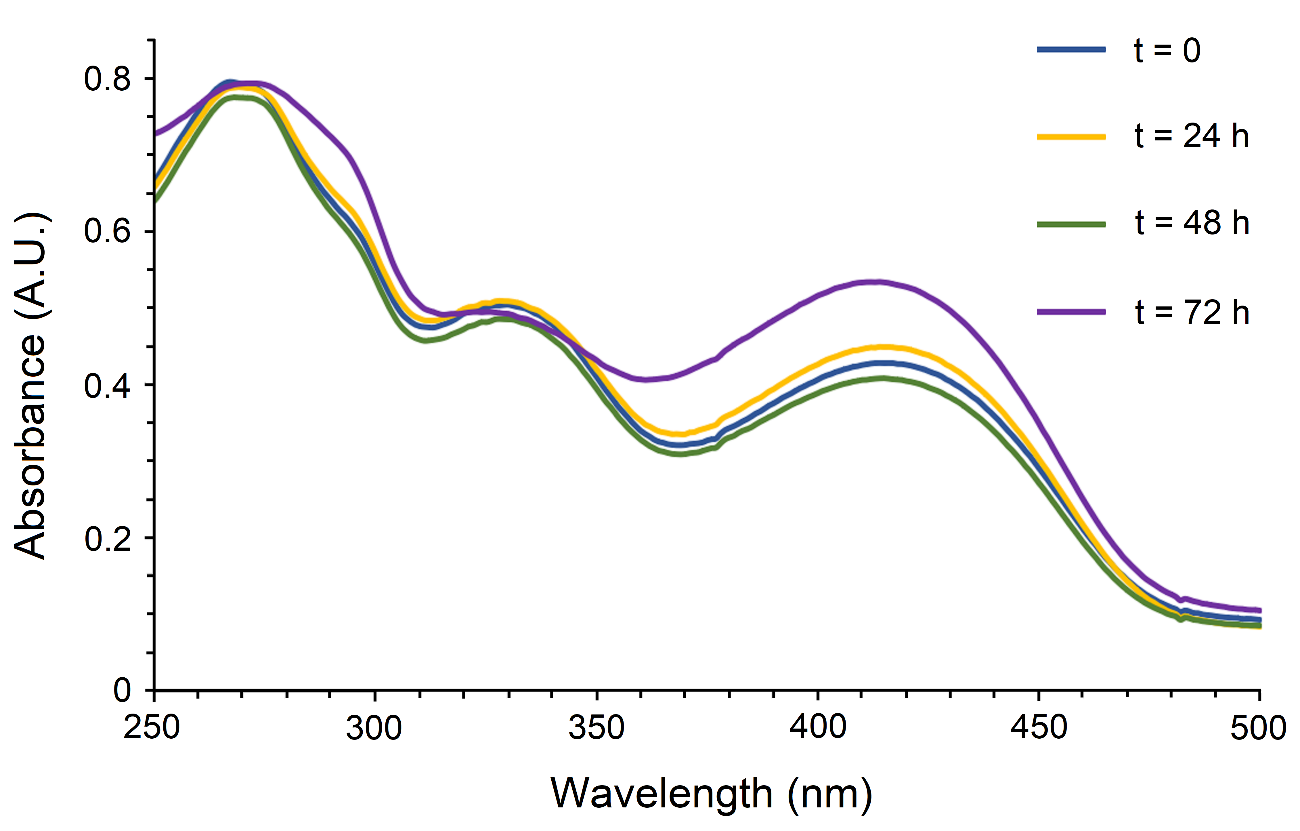


**Fig. S1** Diffuse reflectance spectra of four cellulose papers that were functionalised at different times using the same activated chemosensor solution

*3.2 Characterisation of the Immobilised Chemosensor. Spectral data*


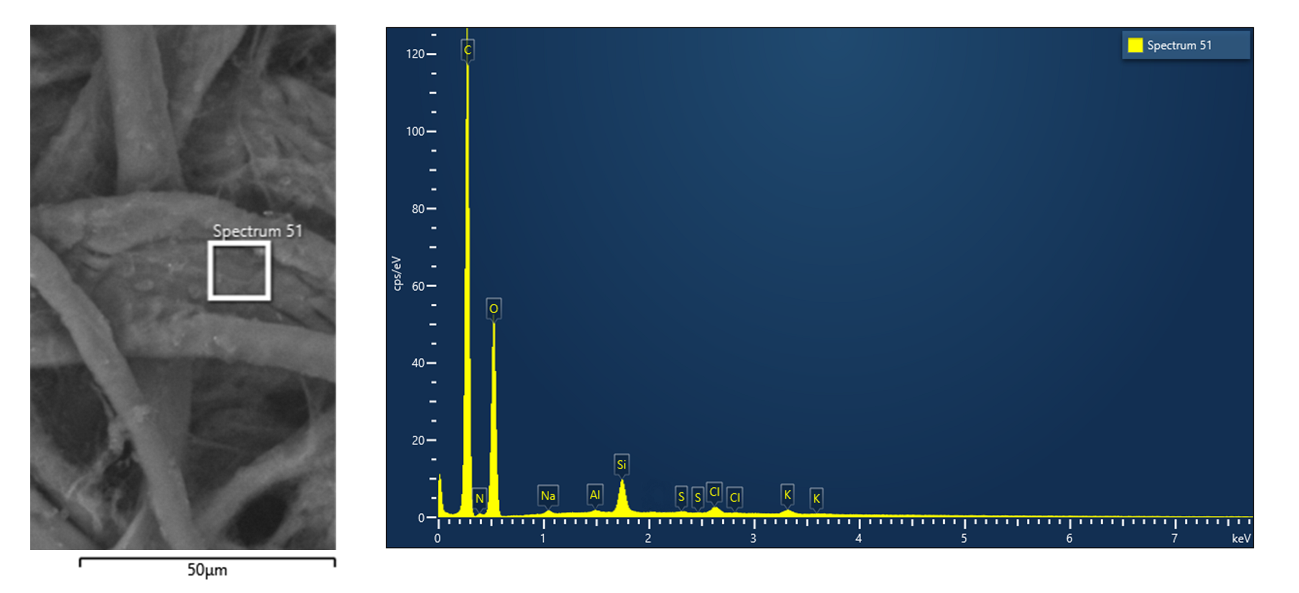


**Fig. S2** SEM image (left) and EDX spectrum (right) of the cellulose paper after immobilisation of the chemosensor.

***3.3*** ***Fluorescence and colorimetric characterisation of the chemosensor. Spectral data***

**
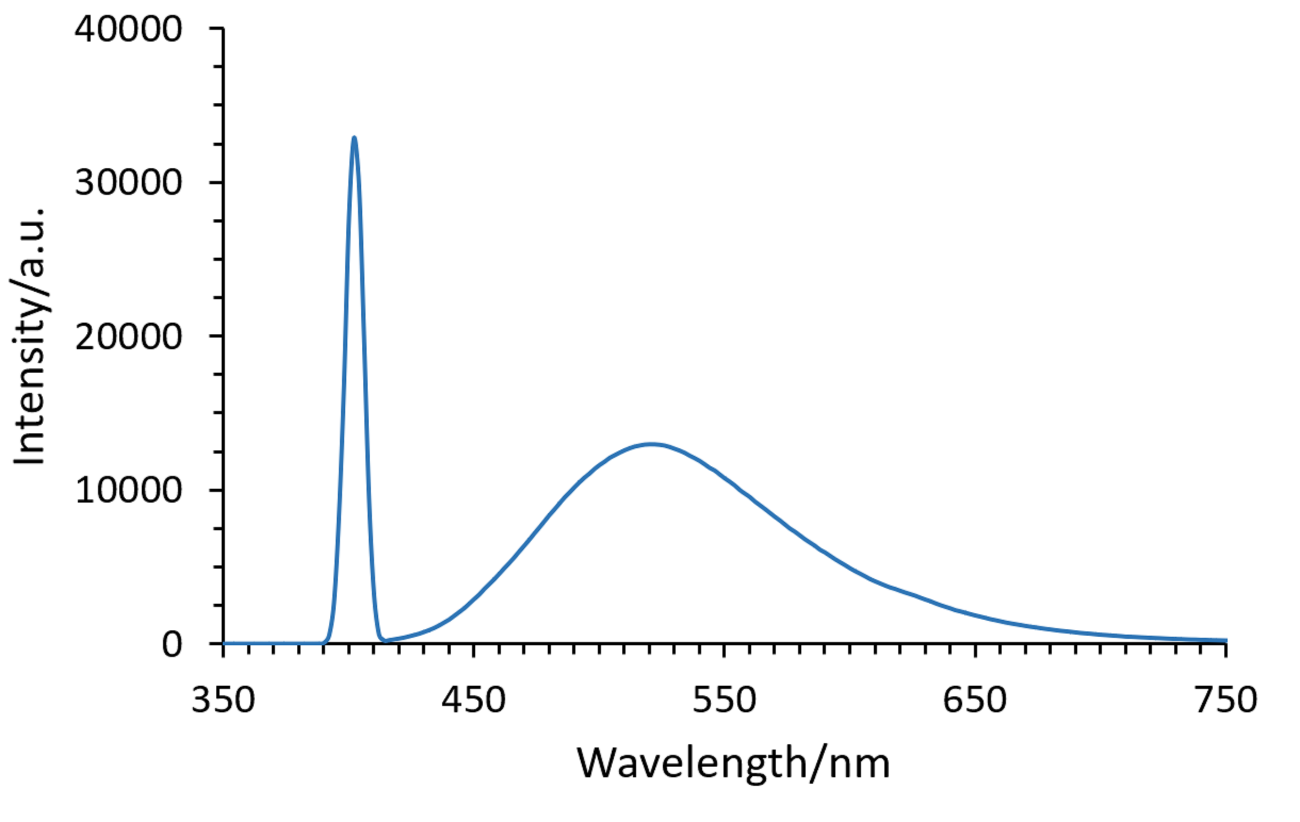
**

**Fig S3**: Fluorescence emission spectrum of the chemosensor (λ_em_ = 520  and λ_ex_= 400 nm).

***3.4*** ***DIC for CuO NPs detection. Spectral data***


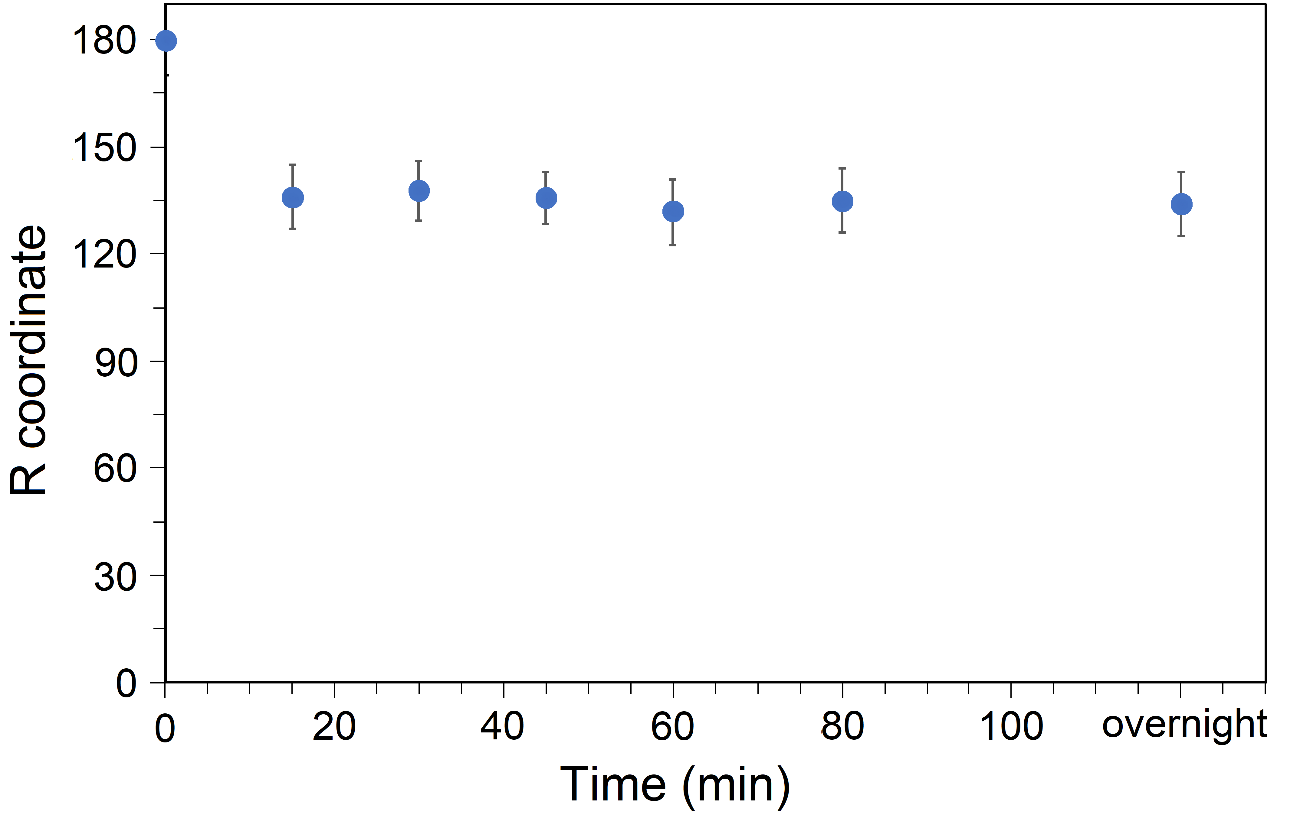


**Fig. S4**. Variation of the R colour coordinate *versus* time

***3.5 DR for CuO NPs detection. Spectral data***


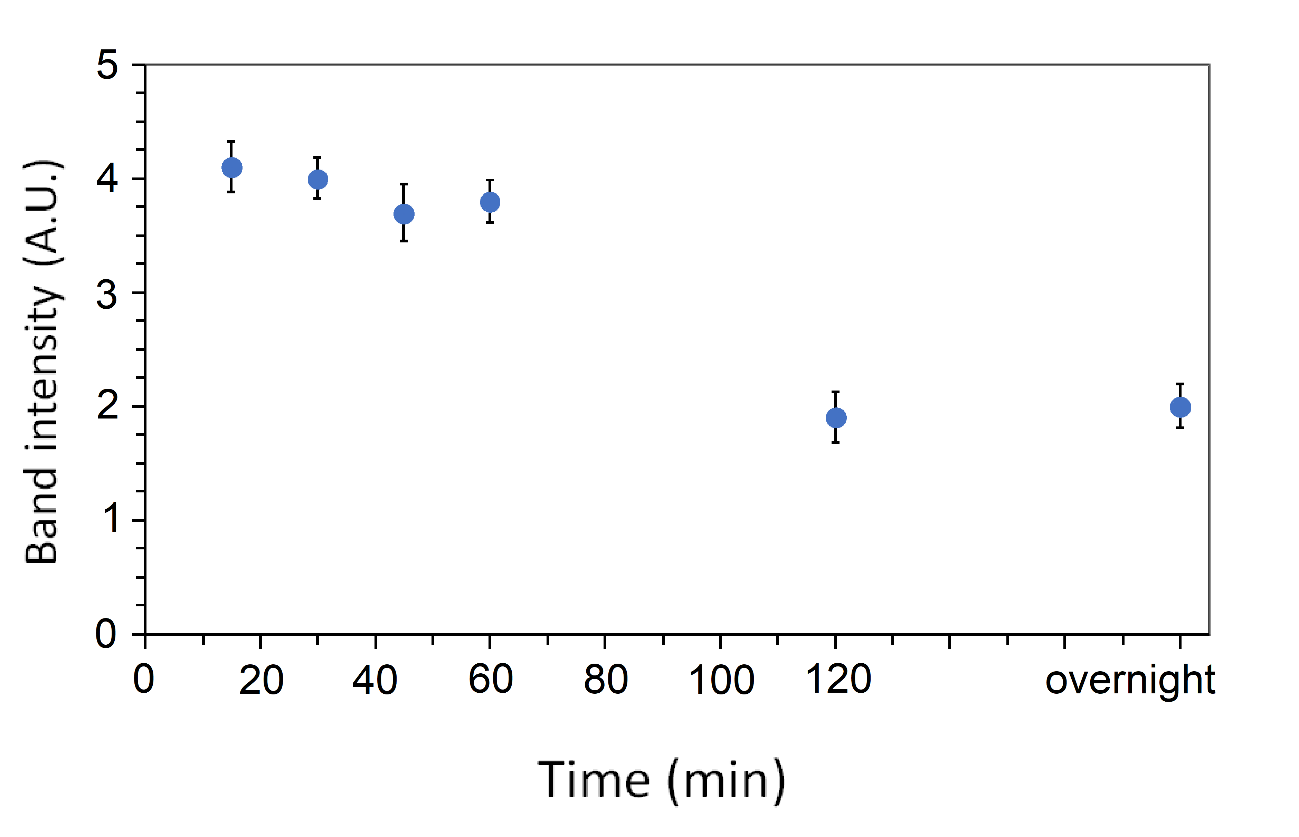


**Fig. S5** Variation of the area under the absorption peak at 270 nm *versus* time
